# Exploring the Fate of Cattle Herds With Inconclusive Reactors to the Tuberculin Skin Test

**DOI:** 10.3389/fvets.2018.00228

**Published:** 2018-09-28

**Authors:** Lucy A. Brunton, Alison Prosser, Dirk U. Pfeiffer, Sara H. Downs

**Affiliations:** ^1^Veterinary Epidemiology, Economics and Public Health Group, Department of Pathobiology and Population Sciences, Royal Veterinary College, University of London, London, United Kingdom; ^2^Data Systems Group, Department of Epidemiological Sciences, Animal and Plant Health Agency, Weybridge, United Kingdom; ^3^College of Veterinary Medicine and Life Sciences, City University of Hong Kong, Kowloon Tong, China; ^4^Epidemiology Group, Department of Epidemiological Sciences, Animal and Plant Health Agency, Weybridge, United Kingdom

**Keywords:** bovine, tuberculosis, SICCT, inconclusive, tuberculin

## Abstract

Bovine tuberculosis (TB) is an important animal health issue in many parts of the world. In England and Wales, the primary test to detect infected animals is the single intradermal comparative cervical tuberculin test, which compares immunological responses to bovine and avian tuberculins. Inconclusive test reactors (IRs) are animals that demonstrate a positive reaction to the bovine tuberculin only marginally greater than the avian reaction, so are not classified as reactors and immediately removed. In the absence of reactors in the herd, IRs are isolated, placed under movement restrictions and re-tested after 60 days. Other animals in these herds at the time of the IR result are not usually subject to movement restrictions. This could affect efforts to control TB if undetected infected cattle move out of those herds before the next TB test. To improve our understanding of the importance of IRs, this study aimed to assess whether median survival time and the hazard of a subsequent TB incident differs in herds with only IRs detected compared with negative-testing herds. Survival analysis and extended Cox regression were used, with herds entering the study on the date of the first whole herd test in 2012. An additional analysis was performed using an alternative entry date to try to remove the impact of IR retesting and is presented in the Supplementary Material. Survival analysis showed that the median survival time among IR only herds was half that observed for clear herds (2.1 years and 4.2 years respectively; *p* < 0.001). Extended Cox regression analysis showed that IR-only herds had 2.7 times the hazard of a subsequent incident compared with negative-testing herds in year one (hazard ratio: 2.69; 95% CI: 2.54, 2.84; *p* < 0.001), and that this difference in the hazard reduced by 63% per year. After 2.7 years the difference had disappeared. The supplementary analysis supported these findings showing that IR only herds still had a greater hazard of a subsequent incident after the IR re-test, but that the effect was reduced. This emphasizes the importance of careful decision making around the management of IR animals and indicates that re-testing alone may not be sufficient to reduce the risk posed by IR only herds in England and Wales.

## Introduction

Bovine tuberculosis (TB) caused by *Mycobacterium bovis* occurs throughout the world, being particularly prevalent in Africa and South America. In Europe, countries that had not achieved Officially Bovine Tuberculosis Free Status (OTF) status in 2016 included Bulgaria, Croatia, Cyprus, Greece, Ireland, Italy, Portugal, Romania, Spain, and the United Kingdom (1). Bovine TB is one of the most important animal health issues in England and Wales, with prevalence of the disease in some parts of England being the highest in the European Union (2). Control of the disease is based on detection and slaughter of infected cattle using immunological testing of cattle herds, restriction of movement from infected herds and carcase inspection of animals at slaughter. Additional testing may be performed in herds perceived to be at risk, e.g., contiguous to an infected herd, or in animals prior to movement. More rigorous testing is applied to herds in which disease is suspected or confirmed.

In England, Defra's strategy for achieving OTF status for England published in 2014 saw the regionalisation of control measures to take account of the spatial heterogeneity of incidence risk (3). The overall incidence rate for England as a whole was 10.2 per 100 herd years at risk in 2016 (4), but this varied considerably across the High Risk (HRA), Edge, and Low Risk (LRA) areas of England [12.8, 3.4, and 0.3 herd years at risk respectively (5)]. In the HRA and Edge area, herds are tested on an annual basis, with herds in some parts of the Edge area being tested every 6 months, whereas in the LRA, herds are tested every 4 years. Tailored control measures are applied to each area in order to meet the objectives of the eradication strategy, which are to achieve OTF status, and more specifically to reduce incidence in the HRA, stop and reverse the spread of disease in the Edge area, and maintain or further reduce incidence in the LRA.

Wales has tested all herds annually since 2008, and in 2016, the TB incidence rate in Wales was 7.0 per 100 herd years at risk (6). Wales has also moved toward a regional approach to TB eradication, by establishing Low, Intermediate, and High TB Areas defined by disease incidence risk. A number of changes to TB control were introduced in October 2017 as part of the Welsh Government's eradication programme (7). In Scotland, which is officially free of tuberculosis, herd-level risk-based surveillance is used for a more targeted approach to routine tests. Herds defined as low-risk are excluded from routine testing.

The primary test used to detect infected animals is the single intradermal comparative cervical tuberculin (SICCT) test, which is based upon injection of bovine and avian tuberculins alongside one another in the skin of the neck. Cattle infected with *M. bovis* tend to show a greater response to bovine tuberculin than avian tuberculin, distinguishing infection with *M. bovis* from infection with other mycobacteria (8). However, while the test is estimated to have high specificity (nearly 100%) (9), the sensitivity of the test at the animal level when using standard interpretation has been estimated to be around 80% but could be as low as 50% (8, 10).

Inconclusive reactors (IRs) to the skin test are defined in England and Wales as animals that demonstrate a reaction to the bovine tuberculin that is less than 4 mm larger than an avian reaction under standard interpretation of the test, or less than 2 mm larger than an avian reaction under severe interpretation. In 2015, there were 2,785 herds in England in which only IRs were detected and which went on to have a re-test, and 21% of these herds had positive reactors (i.e., an incident) at the re-test (5). In Wales, there were 970 IR-only herds of which 21% had an incident at the re-test (6). Animals in these herds at the time of the IR result may be infected, yet the herds will not usually be subject to movement restrictions unless there is a recent history of TB in the herd. In England, 1,420 IRs were slaughtered in 2016 and 13.4% were found to have visible lesions (4). In Wales, 862 IRs were slaughtered in 2016 and 2.9% had visible lesions (6). This could have implications for efforts to control TB if undetected infected cattle move out of those herds over the 60-days period prior to the re-test. This has been demonstrated in Ireland where Clegg et al. (11) reported that between 11.8 and 21.4% of IRs slaughtered before being re-tested were infected with *M. bovis* at post mortem, compared with between 0.13 and 0.22% of animals with a negative SICCT test.

A change in policy for the management of IRs was introduced in England in November 2017. The policy now requires that all IRs in the HRA and Edge Area with a negative result on re-testing must remain restricted for life to the holding in which they were identified. This also applies to IRs in infected herds in the LRA. In comparison, the Welsh eradication programme aims to remove IRs detected in chronically infected herds, under specific circumstances, alongside any reactors. These proactive approaches to managing the risks of IRs are appropriate in light of current knowledge, yet the factors associated with the fate of IR herds are still not well understood. Analysis of 2016 surveillance data has shown that in the HRA and Edge areas of England, herds with a history of TB had a significantly greater risk of having a confirmed incident at the IR retest (4). However, the association between a herd having an IR-only test result and the time to a subsequent incident has not been explored in England and Wales. To improve our understanding of the risk that IRs represent, this study aims to assess whether there are differences in the time to a subsequent incident in herds with only IRs detected compared with herds that test negative at a whole herd test.

## Materials and methods

### Study population and data extraction

A retrospective cohort study followed cattle herds in England and Wales between 1st January 2012 and 31st December 2016. Data describing TB testing and incidence for the study period were obtained from the Animal and Plant Health Agency's Sam database. The study population included all unrestricted herds (TB-free) in the high-risk and edge areas of England and Wales that had a whole-herd type test (WHT) in 2012. This included a small number of routine herd tests (5% of all WHT included) which in some cases might not include all animals in the herd. Herd demographic data, information relating to the first WHT in 2012 and the first subsequent incident (test where reactors were disclosed or infected animals detected at slaughter) were obtained. The number of incidents in the 10 years prior to the 2012 WHT, and the annual rolling county-level incidence at the end of 2012 were also obtained. The dataset was prepared using Microsoft SQL Server 2012 and extracted for cleaning and analysis using Stata 14 (Stata Corporation, College Station, TX, USA).

Herds entered the study on the date of their first WHT in 2012. Herds with a positive test result at the first 2012 WHT, or an incident linked to this test, were excluded. The remaining herds were grouped into two cohorts: those with a clear test result at the 2012 WHT (“clear herds”) and those that had only IRs detected (“IR only herds”). The outcome was defined as a subsequent incident (i.e., reactors detected at a subsequent test or infected animals detected at slaughter) during the follow-up period. Herds were censored either on the date of the test that disclosed an incident or at the end of the study period, whichever was earlier. Herds lost to follow-up due to the closure of the farm contributed time at risk until the date they were archived in Sam. Time was measured in days, but scaled up to years for the analysis.

The hypothesis being tested was that the hazard of a subsequent incident is different between herds in which IRs have been detected and herds which test negative.

### Statistical analyses

Descriptive analyses were performed to examine the number of herds in each cohort (clear herds or IR only herds), and the number of incidents during the follow-up period. The median survival time in years for each cohort was estimated using the Kaplan-Meier method (12). Differences in survival time between the two cohorts were analyzed using the log-rank statistic.

Cox regression was used to examine the association between first WHT status in 2012 and the hazard of a subsequent incident. Other explanatory variables examined for an association with the hazard of a subsequent incident were herd type, herd size, the season in which the 2012 WHT took place, the number of incidents in the previous 10 years, geographical risk area and annual rolling county-level incidence at the end of 2012. These other explanatory variables were then individually added to a model with first WHT status in 2012 to assess whether they resulted in a change in the hazard ratio for the primary exposure. Herd size, the number of incidents in the previous 10 years and county-level incidence were analyzed as both continuous and categorical variables, and those that resulted in the greatest change in the hazard ratio for first WHT status in 2012 were used in the analysis. Efron's method for dealing with ties was used since there were a large number of tied events in the dataset due to the large number of herds and the resolution of the temporal unit (days). All variables associated with the hazard of a subsequent incident with a *p* < 0.20 in univariable analyses were considered for inclusion in a multivariable model.

The multivariable analysis was performed in a stepwise manner with the variable first WHT status in 2012 (“clear” or “IR only”) forced into the model as the primary exposure variable. The outcome variable was occurrence of a subsequent incident. Confounders were then sequentially added to the model in a forward stepwise manner, starting with the variable that resulted in the greatest change in the hazard ratio for first WHT status in the univariable analysis. An interaction between herd type and location was considered. The likelihood ratio test and Akaike's Information Criterion (AIC) were used to compare models (13). Model fit was assessed using Harrell's C concordance statistic and by plotting the Cox Snell residuals and deviance residuals, as recommended by Dohoo et al. (14).

To test the assumption of proportional hazards, a log-minus-log survival plot was generated for first WHT status adjusted for variables included in the final model. The correlation between the Schoenfeld residuals of each variable and transformed time was assessed using the Chi-squared test. A *p* < 0.05 was taken as evidence against the null hypothesis that the hazards were proportional. In addition, graphs of the scaled Schoenfeld residuals over time were plotted for each variable to look for nonlinear relationships between the residuals and time or influential outliers. Interactions between each of the variables and log time were assessed by extending the model to include time varying coefficients using the tvc command in Stata. Model fit could not be assessed using the Cox-snell and deviance residuals after the inclusion of the time-varying coefficients, so models were assessed using the likelihood ratio test and AIC.

An additional analysis was performed using the date of the first subsequent clear herd test after the first WHT as the entry date, thereby excluding herds that were disclosed as infected at the IR retest. The purpose of this was to try to remove the impact of the IR retesting and ensure that all herds were starting out on comparable testing regimes. The results of this analysis are presented in the Supplementary Material.

## Results

### Descriptive analysis

There were 30,600 unrestricted herds that had a WHT in 2012, and overall, the median percentage of animals tested per herd at the first WHT in 2012 was 98%. Of the 30,600 herds, 27,289 (89%) tested negative (clear), and 3,311 (11%) only had IRs (IR only) at the first WHT in 2012. Overall, 30% of herds went on to have a subsequent incident within the follow-up period. A greater percentage of IR only herds went on to have a subsequent incident compared with clear herds (63 and 27% respectively) (Z-test to compare two proportions: *p* < 0.001) (Table 1).

**Table 1:** Number and percentage of herds that had a subsequent incident, stratified by each explanatory variable.

**Variable**	***N***	**Missing**	**Herds with a subsequent incident**		
			***n***	**%**	**95% CI^a^**
**FIRST WHT STATUS IN 2012**					
Clear	27,289	0	7,231	26.5	26.0–27.0
IRs Only	3,311		2,095	63.3	61.6–64.9
**SEASON IN WHICH 2012 WHT TOOK PLACE**					
Spring	9,935	0	2,976	30.0	29.1–30.9
Summer	3,996		1,198	30.0	28.6–31.4
Autumn	7,474		2,253	30.1	29.1–31.2
Winter	9,195		2,899	31.5	30.6–32.5
**NUMBER OF INCIDENTS IN THE PREVIOUS 10 YEARS**					
0–2	27,639	0	7,376	26.7	26.2–27.2
3 or more	2,961		1,950	65.9	64.1–67.5
**GEOGRAPHICAL RISK AREA**					
England high-risk	17,145	0	6,595	38.5	37.7–39.2
England Edge	3,311		636	19.2	17.9–20.5
Wales	10,144		2,095	20.7	19.9–21.5
**ANNUAL ROLLING COUNTY LEVEL INCIDENCE AT THE END OF 2012**					
0–14.6 per 100 herd years at risk	17,431	0	3,983	22.9	22.2–23.5
>14.6 per 100 herd years at risk	13,169		5,343	40.6	39.7–41.4
**HERD TYPE**					
Beef	23,713	0	6,087	25.7	25.1–26.2
Dairy	6,447		3,189	49.5	48.3–50.7
Other	440		50	11.4	8.7–14.7
**HERD SIZE**					
0–10	4,941	1,563	453	9.2	8.4–10.0
11–50	8,697		1,755	20.2	19.4–21.0
51–100	5,488		1,802	32.8	31.6–34.1
101–200	5,164		2,336	45.2	43.9–46.6
201–300	2,196		1,218	55.5	53.4–57.5
>300	2,551		1,700	66.6	64.8–68.4

a *Confidence interval*.

The percentage of herds that suffered a subsequent incident was greater among herds with three or more incidents in the 10 years prior to the 2012 WHT, dairy herds, and increased with herd size (Table 1). In addition, herds appeared to be more likely to have a subsequent incident if they were located in the high-risk area of England and in a county where incidence was greater than the median incidence across all counties at the end of 2012 (Table 1). The percentage of herds that had a subsequent incident did not vary with the season in which the 2012 WHT took place. Among IR only herds, 53% of subsequent incidents were disclosed by an IR retest, whereas among clear herds, 19% of subsequent incidents were disclosed by an IR retest (*Z*-test to compare two proportions: *p* < 0.001). The median number of skin test reactors was lower among incidents disclosed by an IR retest than among incidents disclosed by other tests (0 vs. 1 respectively; Wilcoxon rank-sum test: *p* < 0.001). However, the median numbers of IRs and reactors to the gamma interferon test was zero among incidents disclosed by an IR retest and among incidents disclosed by other tests.

Seven herds were excluded from the analysis as they had an archive date (date herd closed down) that fell before the date of the first WHT in 2012 and they were not tested again within the follow-up period. This left 30,593 herds under observation. There were 9,326 herds with a subsequent incident, which occurred at a median follow-up time of 1.8 years (range: 0.02–4.9), while 21,267 herds were censored at a median follow-up time of 4.5 years (range: 0.03–5.5). There were 3,705 herds lost to follow-up because the business closed down. More clear herds were lost to follow-up (13.1%) than IR only herds (3.8%).

The median survival time among IR only herds was over half that observed for clear herds. Median survival time was also reduced among herds with more than 200 animals, dairy herds, and herds with 3 or more incidents in the previous 10 years (Table 2).

**Table 2:** Median, minimum, and maximum survival time in the clear and IR only cohorts, and by each explanatory variable.

**Variable**	**Level**	**Survival time (years)**		
		**Median**	**Min**	**Max**
First WHT status in 2012	Clear	4.21	0.02	5.46
	IR	2.07	0.02	5.09
Season in which 2012 WHT took place	Spring	4.63	0.02	4.84
	Summer	4.36	0.05	5.46
	Autumn	4.11	0.02	5.28
	Winter	4.08	0.02	5.08
Number of incidents in the previous 10 years	0–2	4.22	0.02	5.46
	3 or more	2.36	0.02	5.42
Geographical risk area	England high-risk	4.07	0.02	5.28
	England Edge	4.26	0.05	5.46
	Wales	4.31	0.12	5.19
Annual rolling county level incidence at end of 2012	0–14.6 per 100 herd years at risk	4.27	0.04	5.46
	>14.6 per 100 herd years at risk	4.31	0.12	5.19
Herd type	Beef	4.23	0.02	5.46
	Dairy	3.76	0.02	5.22
	Other	4.25	0.18	4.99
Herd size	0–10	4.34	0.03	5.25
	11–50	4.36	0.05	5.42
	51–100	4.25	0.06	5.46
	101–200	4.11	0.02	5.22
	201–300	3.40	0.02	5.15
	>300	2.57	0.02	5.24

**Figure 1:**
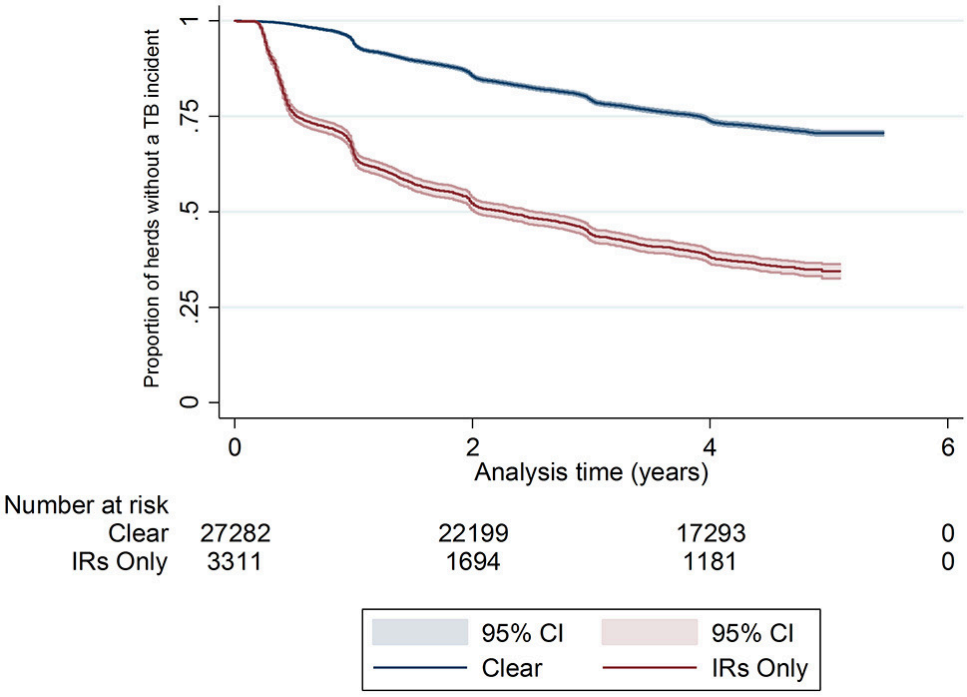
Kaplan-Meier survival estimates for herds according to first WHT status in 2012.

**Figure 2:**
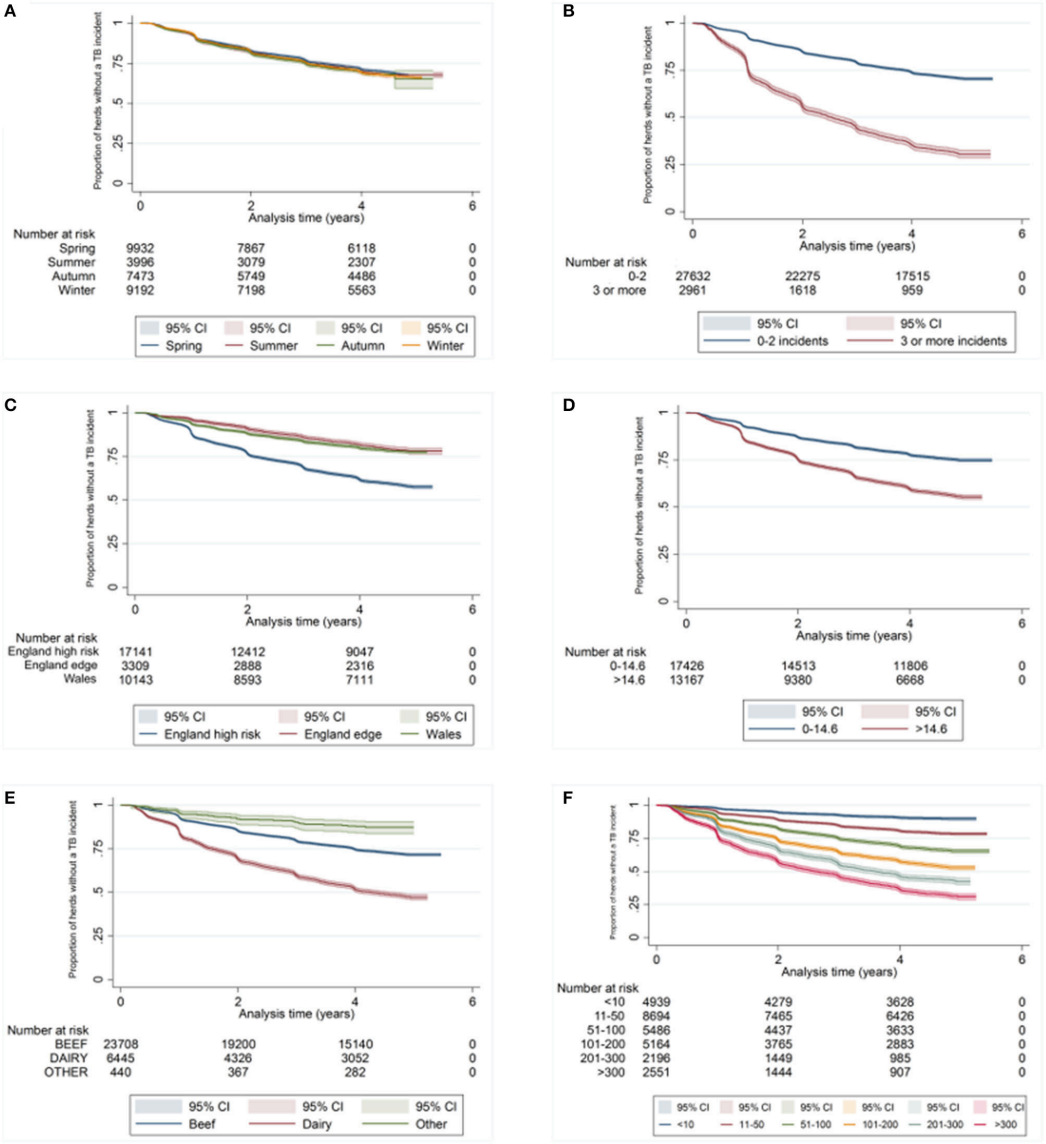
Kaplan-Meier survival estimates for herds according to season in which 2012 WHT took place **(A)**, number of incidents in the previous 10 years **(B)**, geographical risk area **(C)**, annual rolling county level TB incidence at end of 2012 **(D)** herd type **(E)**, and herd size **(F)**.

There was a difference in the survival functions of the clear and IR only cohorts (Figure 1) and this observation was supported by the results of the log-rank test (Table 3). Significant differences in survival were also observed between herds grouped according to their TB history, geographical area, county level incidence, production type, and size (Figures 2B–F). The survival of herds did not appear to vary according to the season in which their 2012 WHT took place (Figure 2A), although the log-rank test indicated there was some evidence of a difference (*p* = 0.04).

**Table 3:** Results of the log-rank tests for equality of survivor functions.

**Variable**	**Chi-squared**	***P*-value**
First WHT status in 2012	3,008.9	<0.001
Season in which 2012 WHT took place	8.51	0.037
Number of incidents in the previous 10 years	2,635.7	<0.001
Geographical risk area	1,238.86	<0.001
Herd type	1,535.93	<0.001
Herd size	4,388.12	<0.001
Annual rolling county level incidence at end of 2012	1,207.05	<0.001

### Assessment of the hazard of subsequent incidents among clear and IR only herds

A Cox regression was performed to assess the hazard of a subsequent incident within the two cohorts. There were strong associations between each of the explanatory variables and the hazard of subsequent incidents in the univariable analysis (Table 4). Factors found to be associated with increased relative hazard of a subsequent incident were having an IR only test result at the 2012 WHT, having the first 2012 WHT in autumn or winter compared with spring, a recent history of TB, increased county-level incidence, being a dairy herd (compared to a beef herd), and increasing herd size. Herds in the edge area of England, and those in Wales, had a reduced incidence rate when compared to the high-risk area of England. Herds classed as production type “other” also had a reduced incidence rate compared with beef herds (Table 4).

**Table 4:** Results of the univariable Cox regression analysis of factors associated with the rate of subsequent incidents.

**Variable**	**Level**	**HR^a^**	**95% CI^b^**		***P*-value**
First WHT status in 2012	Clear	*1.00*			
	IRs only	3.58	3.41	3.76	<0.001
Season in which first WHT took place	Spring	*1.00*			
	Summer	1.06	0.99	1.13	0.105
	Autumn	1.08	1.02	1.14	0.007
	Winter	1.06	1.01	1.11	0.031
Number of incidents in the previous 10 years	<3	*1.00*			
	3 or more	1.50	1.49	1.52	<0.001
Geographical risk area	England high risk	*1.00*			
	England Edge	0.43	0.40	0.47	<0.001
	Wales	0.47	0.44	0.49	<0.001
Annual rolling county level incidence at end of 2012	0–14.6 per 100 herd years at risk	*1.00*			
	>14.6 per 100 herd years at risk	1.07	1.07	1.07	<0.001
Herd type	Beef	*1.00*			
	Dairy	2.26	2.16	2.36	<0.001
	Other	0.44	0.33	0.58	<0.001
Herd size	1–10	*1.00*			
	11–50	2.21	1.99	2.45	<0.001
	51–100	3.82	3.44	4.23	<0.001
	101–200	5.74	5.19	6.35	<0.001
	201–300	7.71	6.92	8.59	<0.001
	>300	10.49	9.45	11.63	<0.001

a *Hazard ratio*.

b *Confidence interval*.

*Ratios in italics represent the reference groups*.

**Figure 3:**
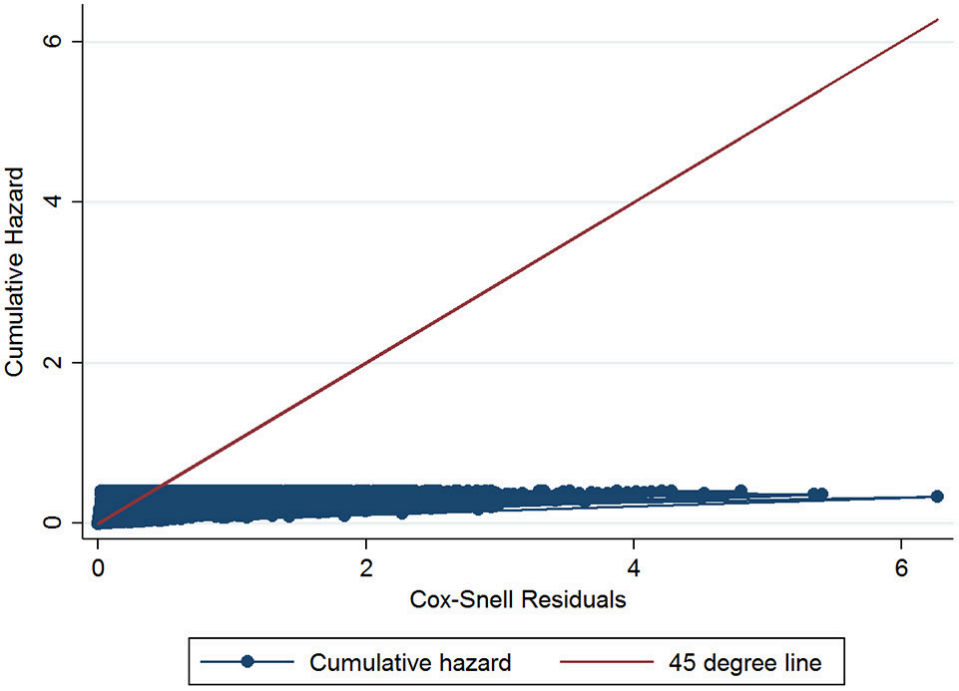
Plot of Cox-snell residuals for the initial Cox regression.

**Figure 4:**
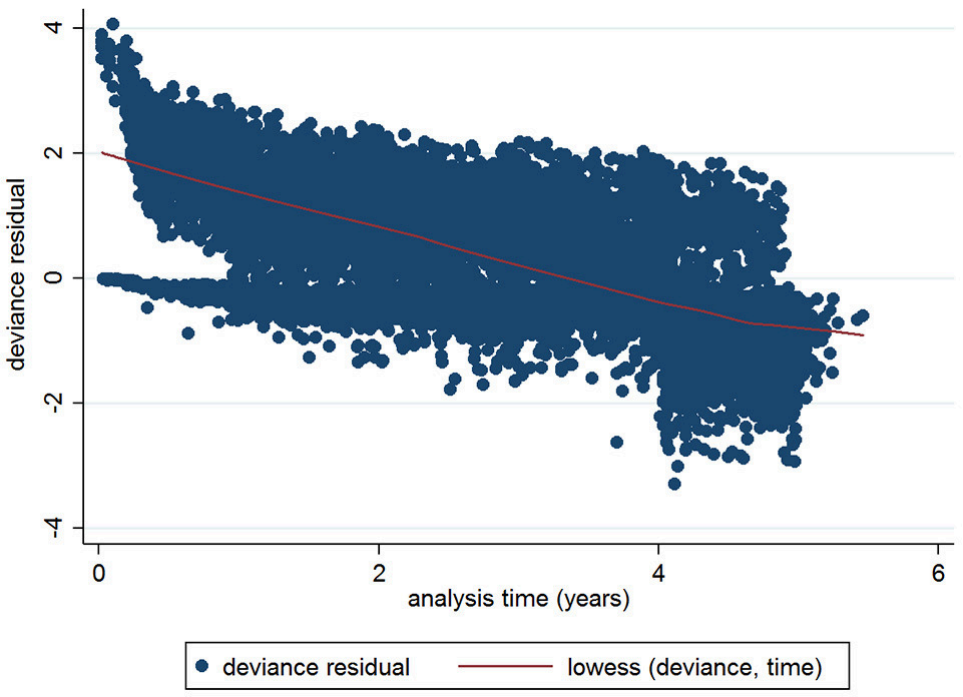
Plot of deviance residuals for the initial Cox regression.

**Figure 5:**
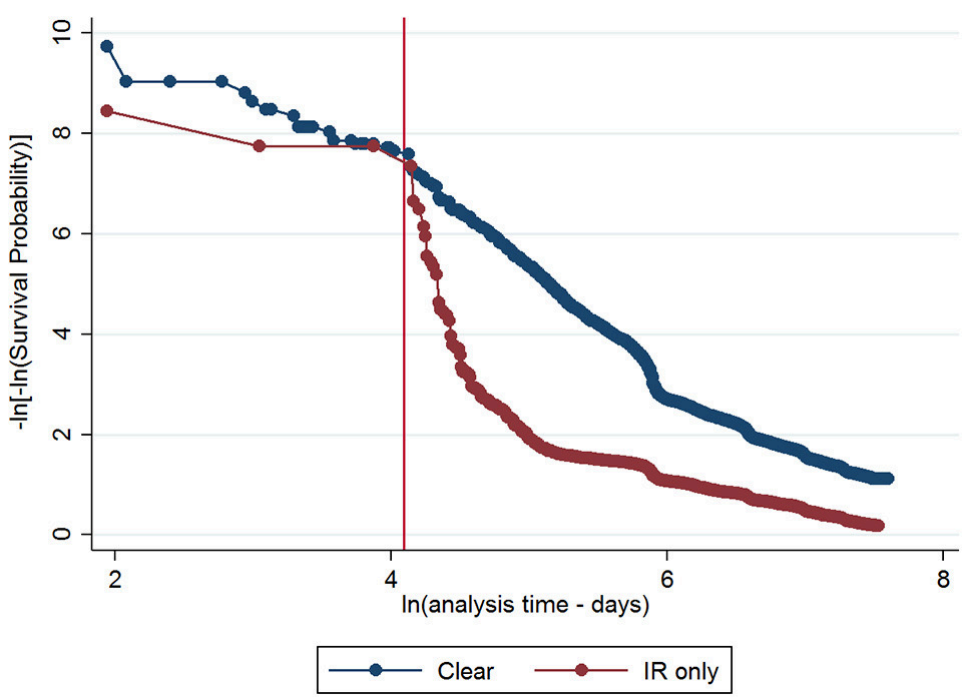
Log-minus-log survival plot for first WHT status adjusted for herd size, the number of incidents in the 10 years before the first WHT in 2012, herd type, county level TB incidence and geographical risk area. A reference line has been added to indicate the change in the HR at 60 days.

The initial multivariable Cox regression model included first WHT status in 2012, herd size, the number of incidents in the 10 years before the first WHT in 2012, herd type, county-level TB incidence and geographical risk area. The plot of the Cox-Snell residuals (Figure 3) indicated that the model was a poor fit, and the plot of the deviance residuals over time (Figure 4) revealed a number of observations that were not well fit by the model, particularly those herds with the shortest survival time. However, the Harrell's C statistic was 0.75 indicating that the model correctly predicted the sequence of two observed failures 75% of the time. Assessment of the proportionality of the hazards using the log-minus-log plot (Figure 5) indicated that the ratio of hazards varied over time. The Chi-squared test of the correlation between the Schoenfeld residuals of each variable and transformed time generated a *p* < 0.05 for all variables except local incidence, indicating that the proportional hazards assumption had been violated. The log-minus-log plot illustrated a change in the ratio of hazards around 60 days, which correlated with the timing of IR retests. This indicated that an analysis of the time to a subsequent incident may not be appropriate given the differences in follow-up testing between the cohorts, and that time varying coefficients should be included to model interactions between the explanatory variables and time.

**Figure 6:**
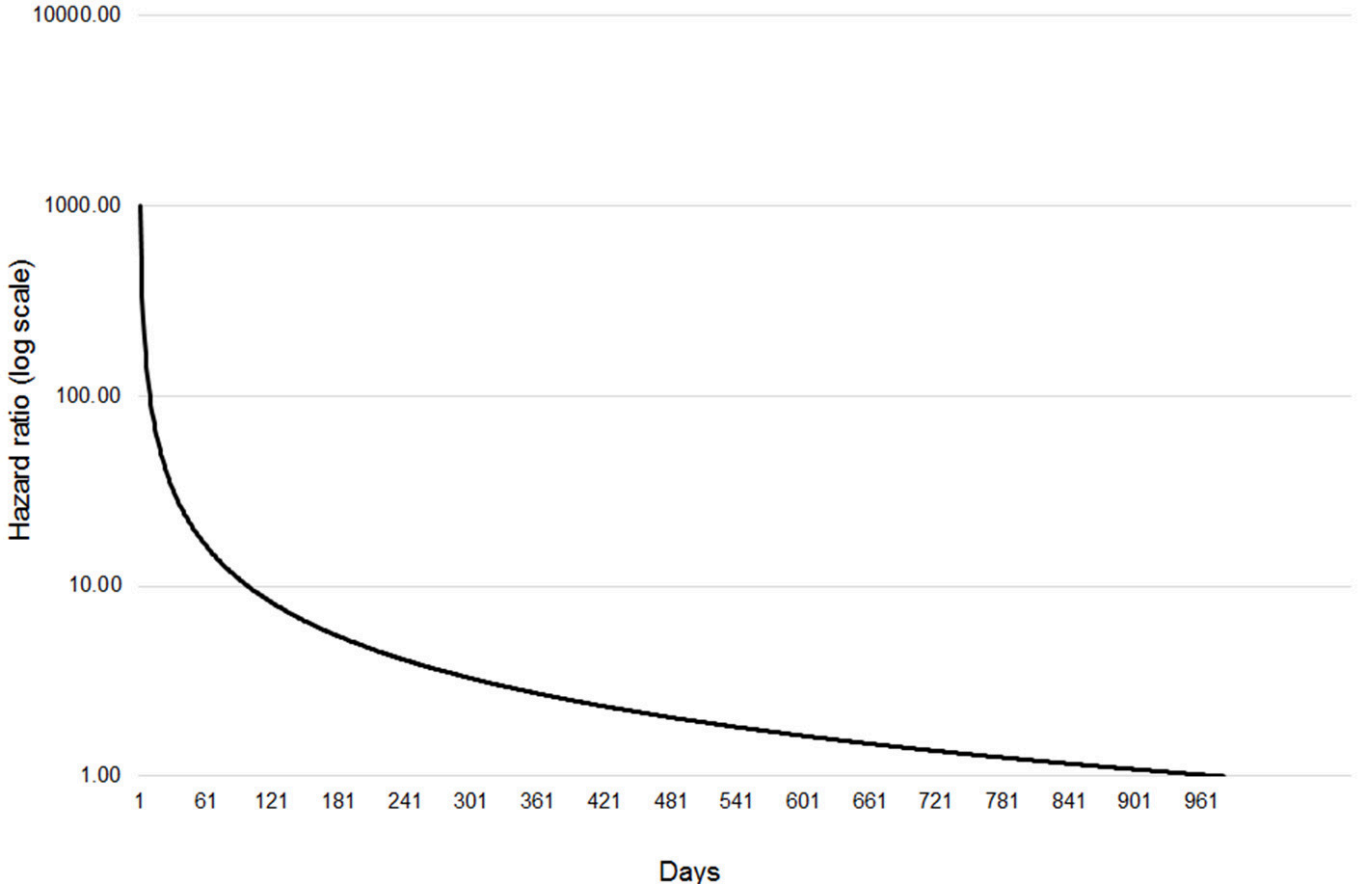
Change in relative hazard over time amongst IR only herds compared with clear herds, adjusted for herd size, the number of incidents in the 10 years before the first WHT in 2012, herd type, county level TB incidence and geographical risk area, and interactions between time and first WHT status, herd size, the number of incidents in the 10 years before the first WHT in 2012, herd type, and geographical risk area.

The final extended Cox regression model contained first WHT status, herd size, recent history of TB, herd type, local incidence and geographical risk area, and included interactions between time and first WHT status, herd size, TB history, risk area and herd type. The relative hazard of having a subsequent incident was 2.7 times greater among herds that were IR only at the 2012 WHT compared with herds that had a clear test result (after adjusting for herd size, testing following the 2012 WHT, recent history of TB, herd type, local incidence and geographical risk area) (Table 5). The interaction with time indicated that the increased relative hazard of having a subsequent incident among IR only herds decreased by 63% each year. This means that according to the model, the relative hazard of 2.7 in year one is reduced to 1.34 in year two, and drops to 0.89 by year three. This change in relative hazard over time is presented in Figure 6. This shows that the effect disappears (i.e., the relative hazard = 1) by around 970 days, or 2.7 years.

**Table 5:** Multivariable extended Cox regression model of factors associated with a subsequent incident amongst clear and IR only herds, including time varying coefficients.

**Variable**	**Level**	**HR^a^**	**95% CI^b^**		***P*-value**
**MAIN COVARIATES**					
First WHT status in 2012	Clear	*1.00*			
	IRs only	2.69	2.54	2.84	<0.001
Herd size	1–10	*1.00*			
	11–50	1.92	1.70	2.17	<0.001
	51–100	3.00	2.66	3.39	<0.001
	101–200	3.93	3.49	4.43	<0.001
	201–300	4.65	4.09	5.30	<0.001
	>300	6.18	5.45	7.02	<0.001
Number of incidents in the previous 10 years		1.19	1.17	1.21	<0.001
Herd type	Beef	*1.00*			
	Dairy	0.98	0.93	1.04	0.547
	Other	0.61	0.45	0.82	0.001
Annual rolling county level incidence at end of 2012	0–14.6 per 100 herd years at risk	*1.00*			
	>14.6 per 100 herd years at risk	1.05	1.05	1.06	<0.001
Geographical risk area	England high risk	*1.00*			
	England Edge	0.90	0.80	1.02	0.088
	Wales	0.80	0.75	0.86	<0.001
**TIME-VARYING COEFFICIENTS**					
First WHT status in 2012	Clear	*1.00*			
	IRs only	0.37	0.34	0.39	<0.001
Herd size	1–10	*1.00*			
	11–50	1.20	1.05	1.38	0.008
	51–100	1.26	1.10	1.44	0.001
	101–200	1.32	1.16	1.51	<0.001
	201–300	1.46	1.26	1.69	<0.001
	>300	1.40	1.21	1.61	<0.001
Number of incidents in the previous 10 years		1.02	1.01	1.04	0.008
Geographical risk area	England high risk	*1.00*			
	England Edge	1.04	0.93	1.17	0.464
	Wales	0.88	0.83	0.94	<0.001
Herd type	Beef	*1.00*			
	Dairy	1.14	1.07	1.21	<0.001
	Other	0.62	0.44	0.88	0.007

a *Hazard ratio*.

b *Confidence interval*.

*Ratios in italics represent the reference groups*.

## Discussion

Understanding the level of infection that could be present among IRs is important for directing control measures. In Ireland, Clegg et al. (11) found that IRs that passed the IR retest and then moved herds within 6 months were 12 times more likely to have a positive result at the next test, or have lesions detected at slaughter, compared to all animals in Ireland. Our analysis has shown that the time interval before a new TB incident in IR only herds was around half that of herds with a negative whole herd test; and that the hazard of a subsequent incident was 2.7 times greater for IR only herds compared with clear herds after accounting for the influence of traditionally accepted drivers of TB. This difference in hazard decreased over time by 63% per year.

The number of incidents in the 10 years prior to the study was consistently associated with an increase in the hazard of a subsequent incident. This is in agreement with other studies where TB history has been identified as a risk factor for future incidents (15–17). Herd size has frequently been associated with increased disease risk (1, 15, 18, 19), but this association can be difficult to interpret. An effect of increasing herd size may simply reflect changes in other risk factors related to farm management, or it may have implications on the sensitivity and specificity of the test at herd level (20).

Dairy herds located within areas subject to badger culling in England were shown to have a greater risk of TB than beef herds in the same areas (21). It has also been shown in separate analyses for England and Wales that the effect of herd type is reduced after adjusting for herd size and location (4, 6). In this study, there was no difference in the rate of subsequent incidents among dairy compared with beef herds, after adjusting for herd size, location and other factors that were not included in the country-level analyses described above (4, 6). However, the time-varying coefficient for herd type was significant for dairy. This suggests that the hazard of a subsequent incident among dairy herds increases by 14% each year. This may be related to the longer life expectancy of dairy cattle compared to beef cattle, meaning that dairy cattle are at risk of exposure to TB for longer than beef cattle (21, 22). Both O'Hagan et al. (23) and Downs et al. (24) have shown that dairy SICCT reactors are less likely to have visible lesions than beef reactors, which could indicate that infected dairy cattle are detected through SICCT surveillance earlier than beef cattle. Therefore, one might expect IRs from beef herds to pose a higher future risk than IRs from dairy herds.

Increased county-level incidence was associated with an increased hazard of a subsequent incident, and herds in the edge area of England and in Wales had a reduced hazard compared with herds in the high risk area of England. Olea-Popelka et al. (15) and Green et al. (25) both showed that increased local prevalence of TB is associated with an increased risk of infection. Johnston et al. (26) found regional variation in risk factors for TB incidents, and Brunton et al. (27) reported spatial heterogeneity in the factors associated with the spread of endemic TB. The significant time-varying coefficient for Wales is interesting, and indicates that the hazard for herds in Wales reduces over time. This was not seen for herds in England, so could be related to differing policies on IRs in the two countries.

The TB testing regime in England and Wales is determined by factors such as location, animal movements and disease history. As such, it varies considerably between herds across both cohorts. However, there are also structural differences in the data due to the TB control policy. IRs have a subsequent test following disclosure of IRs, which does not take place in herds where all the cattle tested negative to the whole herd test. This increases the probability of IR-only herds having a subsequent incident compared with herds that tested clear, since increased testing increases the chances of detecting disease. This is further complicated by the fact that animals that have a second IR test result at the follow up test will automatically be classified as reactors. This means that there is a bias toward detecting cases within the IR only cohort. Unfortunately, the structure of the data did not allow the analysis of individual test data for each herd to explore the impact of this further. Instead, the time-varying coefficients were included to model how the relative hazard of a subsequent incident amongst IR only herds compared with clear herds varied over time. A reduction in the hazard ratio over time was observed, which indicates that the hazard for IR only herds becomes comparable to that of clear herds after around two and a half years. If the effect of re-testing was the only reason that IR only herds had a greater hazard of a subsequent incident, then we would expect the hazard ratio to reach 1.0 after the 60 days retest. The fact that it takes over 2 years to reach 1.0 suggests that the hazard of a subsequent TB incident is still higher among IR only herds than herds that tested negative to a whole herd test once the effect of re-testing has been removed.

An additional analysis was performed to try to remove the impact of the IR re-testing by ensuring that all herds were starting out on comparable testing regimes, and the results of this analysis are presented in the Supplementary Material. The results of this additional analysis indicate that there is still a significantly greater hazard of a subsequent incident amongst IR only herds compared with clear herds, but that this is reduced once the effect of re-testing is removed. This aligns with the finding that the hazard ratio is still greater than 1.0 after the 60 days re-test has passed. However, the additional analysis needs to be interpreted cautiously as the sample size for the IR cohort was reduced by almost half (46%) due to missing or inaccurate values within the subsequent clear test variable used as the new entry date. The clear herd cohort was less affected by missing values (15%). This introduces a considerable bias to the additional analysis and makes it difficult to draw firm conclusions from this about the fate of IR only herds compared to clear herds after they get through the IR testing regime.

There is potential for the misclassification of IRs due to the imperfect test for TB. The influence of disease prevalence on the predictive value of the test also introduces the potential for misclassification across risk areas. For example, the low positive predictive value of the test when prevalence is low means that IRs in the low-risk areas may be false positives, while the low negative predictive value of the test when prevalence is high means that IRs in high-risk areas may be false negatives. Even if perfect classification were possible, the nature of IRs is that their infection status is uncertain. They may be uninfected animals that have been exposed to other mycobacteria, or they may be infected animals that do not respond adequately to the test due to factors such as immunosuppression or co-infection (8). This uncertainty makes managing the potential risk that IRs pose challenging, and highlights the need for evidence to understand this risk.

The finding that the hazard of a subsequent incident reduces over time among IR only herds indicates that the policy in England and Wales for dealing with IRs is having an effect. However, these herds still appear to be at greater risk of having an incident after the IR re-testing regime. This could reflect that the testing is not removing all potentially infected animals from the herd, or there may be other factors which put these herds at a greater risk of having a TB incident that we have yet to understand. This is important information for both policy makers in England and Wales, and those in other countries looking to learn from the English and Welsh experience in tackling bovine TB. The evidence from this analysis suggests that the new policy decision in England, restricting IRs with a negative re-test to the herd in which they were detected for life, should help reduce any residual risk associated with an IR for disease spread. This approach has been implemented in Ireland since 2012 (28) following the analysis of the fate of IRs by Clegg et al. (29).

The present study has shown that the hazard of a subsequent TB incident is greater among IR only herds than herds that tested negative to a whole herd test, and that the hazard ratio decreases over time, but remains greater than 1.0 after the IR re-testing regime. This emphasizes the importance of careful decision making around the management of IR animals and indicates that re-testing alone may not be sufficient to reduce the risk posed by IR only herds. Further characterisation of IRs is needed to determine whether the differences observed here are related to management or biological factors. This may be best achieved through an animal-level analysis so that the risk of retaining individual IR animals in a herd in England and Wales can be understood. Our findings correlate with the Irish findings, indicating that the risks of IRs are unlikely to be country and context specific. This provides further evidence of the risk that IRs pose for the spread of TB, which can support the development of policies in other countries relating to the management of IRs.

## Author contributions

LB designed the study, performed the analysis, and drafted the manuscript in part fulfillment of the requirements for the degree of Master of Science in Veterinary Epidemiology at the Royal Veterinary College, University of London. AP generated the dataset and edited the manuscript. DP and SD provided advice on study design and analysis, made additions to the text, and edited the manuscript.

### Conflict of interest statement

The authors declare that the research was conducted in the absence of any commercial or financial relationships that could be construed as a potential conflict of interest.
