# Supplementary material for: Exploring the Fate of Cattle Herds With Inconclusive Reactors to the Tuberculin Skin Test
Source: Front Vet Sci. 2018 Sep 28;5:228. doi: 10.3389/fvets.2018.00228 (PMC6173137; doi:10.3389/fvets.2018.00228)
Supplement: Supplementary file 1 [file Data_Sheet_1.docx]

Supplementary Material

Exploring the fate of cattle herds with inconclusive reactors to the tuberculin skin test

Brunton, Lucy A.^*^, Prosser, Alison, Pfeiffer, Dirk U. & Downs, Sara. H.

*** Correspondence:** Dr Lucy A. Brunton: lbrunton@rvc.ac.uk

Additional analysis using revised entry date

Inconclusive reactors (IRs) to the tuberculin skin test are subject to a retest at around 60 days, which does not take place in herds where all the cattle tested negative to the whole herd test. The additional testing in the IR cohort increases the probability of IR-only herds having a subsequent incident compared with herds that tested clear, since increased testing increases the chances of detecting disease. In addition, animals that have a second IR test result at the retest will automatically be classified as reactors. This means that there is a bias towards detecting cases within the IR only cohort. In an attempt to mitigate this, the analysis described in the accompanying manuscript was repeated using a revised entry date as described below.

# Methods

Herds entered the study on the date of their first subsequent clear test following the first whole herd test (WHT) in 2012. Herds where the first subsequent clear test fell on or after the date of a subsequent incident were removed from the analysis as these incidents were considered to be linked to the first WHT. The remaining herds were grouped into two cohorts: those with a clear test result at the 2012 WHT (“clear herds”) and those that had only IRs detected (“IR only herds”). Cases were defined as herds that had a subsequent incident (i.e. reactors detected at a subsequent test) during the follow-up period. Herds were censored either on the date of the test that disclosed an incident or at the end of the study period, whichever was earlier. Herds lost to follow-up due to the closure of the farm contributed time at risk until the date they were archived in the Sam database. The unit of measurement for time was days, scaled to years for the analysis.

The hypothesis being tested was that when on comparable testing regimens, the hazard of a subsequent incident is different between herds in which IRs have been detected and herds which test negative.

Descriptive analysis, survival analysis and Cox regression analysis were performed in the same way as described in the accompanying manuscript.

# Results

## Descriptive analysis

Out of the study sample of 30,600 herds, there were 1,545 herds (5%) for which no subsequent clear test date was recorded. The reasons for no subsequent clear test date being recorded were investigated and the following reasons were identified:

A clear test date existed, but no animals were recorded as being tested on that date

The herd closed down before it was due to be tested again

The herd had had IR only results between the first WHT and a subsequent incident or leaving the study

In addition, for some herds there was no obvious reason why a subsequent clear test date was not recorded. The percentage of herds without a subsequent clear test date were compared across the levels of the outcome variable (a subsequent incident), the primary exposure variable (first WHT status) and for each of the other explanatory variables included in the analysis (Supplementary Table 1). Herds were more likely to have no subsequent clear test date recorded if they had no subsequent incident, tested clear at the first WHT in 2012, were of a herd type classed as “other”, were located in the edge area of England, and had less than 50 animals.

**Supplementary Table 1.** The percentage of herds without a subsequent clear test date stratified by each variable

| **Variable** | **Level** | **Subsequent clear test date not recorded (%)** |
| --- | --- | --- |
| Subsequent incident | Yes | 0.6 |
|  | No | 7.0 |
| First WHT status | Clear | 5.4 |
|  | IR only | 2.5 |
| Herd type | Beef | 5.4 |
|  | Dairy | 3.6 |
|  | Other | 9.8 |
| Geographical risk area | England high-risk | 5.3 |
|  | England edge | 6.5 |
|  | Wales | 4.1 |
| Annual rolling county level incidence at end of 2012 | 0-14.6 per 100 HYR^a^ | 5.0 |
|  | >14.6 per 100 HYR^a^ | 5.1 |
| Herd size | <10 | 8.6 |
|  | 11-50 | 2.8 |
|  | 51-100 | 1.5 |
|  | 101-200 | 1.1 |
|  | 201-300 | 1.8 |
|  | >300 | 1.8 |

There were 4,134 herds where the first subsequent clear test fell on or after the date of a subsequent incident (i.e. the exit date). These herds were removed from the analysis along with the 1,545 herds where the subsequent clear test date was missing. A greater proportion of IR only herds were removed than clear herds (46% removed vs 15% removed). This left 24,921 herds under observation, of which 5,139 herds had a subsequent incident. The median time to failure among IR only herds was similar to that observed for clear herds. Median time to failure was slightly reduced among herds with three or more incidents in the previous 10 years, and herds with more than 300 animals (Supplementary Table 2).

**Supplementary Table 2.** Median, minimum and maximum time to failure in the clear and IR only cohorts, and by each explanatory variable (N=24,921)

| **Variable** | **Level** | **Time to failure (years)** | | |
| --- | --- | --- | --- | --- |
|  |  | **Median** | **Min** | **Max** |
| First WHT status in 2012 | Clear | 3.27 | 0.02 | 5.03 |
|  | IR | 3.05 | 0.02 | 4.79 |
| Season in which 2012 WHT took place | Spring | 3.67 | 0.02 | 4.79 |
|  | Summer | 3.38 | 0.02 | 4.54 |
|  | Autumn | 3.14 | 0.04 | 5.03 |
|  | Winter | 3.61 | 0.04 | 4.99 |
| Number of incidents in the previous 10 years | <3 | 3.29 | 0.02 | 5.03 |
|  | 3 or more | 2.88 | 0.04 | 4.94 |
| Geographical risk area | England high-risk | 3.20 | 0.02 | 4.98 |
|  | England Edge | 3.10 | 0.02 | 4.99 |
|  | Wales | 3.50 | 0.04 | 5.03 |
| Annual rolling county-level incidence at end of 2012 | 0-14.6 per 100 HYR^a^ | 3.31 | 0.02 | 5.03 |
|  | >14.6 per 100 HYR^a^ | 3.19 | 0.02 | 4.98 |
| Herd type | Beef | 3.29 | 0.04 | 5.03 |
|  | Dairy | 3.10 | 0.02 | 4.96 |
|  | Other | 3.32 | 0.07 | 4.94 |
| Herd size | 0-10 | 3.36 | 0.06 | 4.85 |
|  | 11-50 | 3.42 | 0.04 | 4.97 |
|  | 51-100 | 3.33 | 0.10 | 4.96 |
|  | 101-200 | 3.19 | 0.02 | 4.98 |
|  | 201-300 | 3.06 | 0.16 | 5.03 |
|  | >300 | 2.91 | 0.02 | 4.99 |

There was a difference in the survival functions of the clear and IR only cohorts (Supplementary Figure 1) and this observation was supported by the results of the log-rank test (p<0.001) (Supplementary Table 3). Significant differences in survival were also observed between herds grouped according to their TB history, between herds in different geographical areas, between herds grouped according to their production type and size, and between herds group by the county-level incidence. The survival of herds did not appear to vary according to the season in which their 2012 WHT took place.


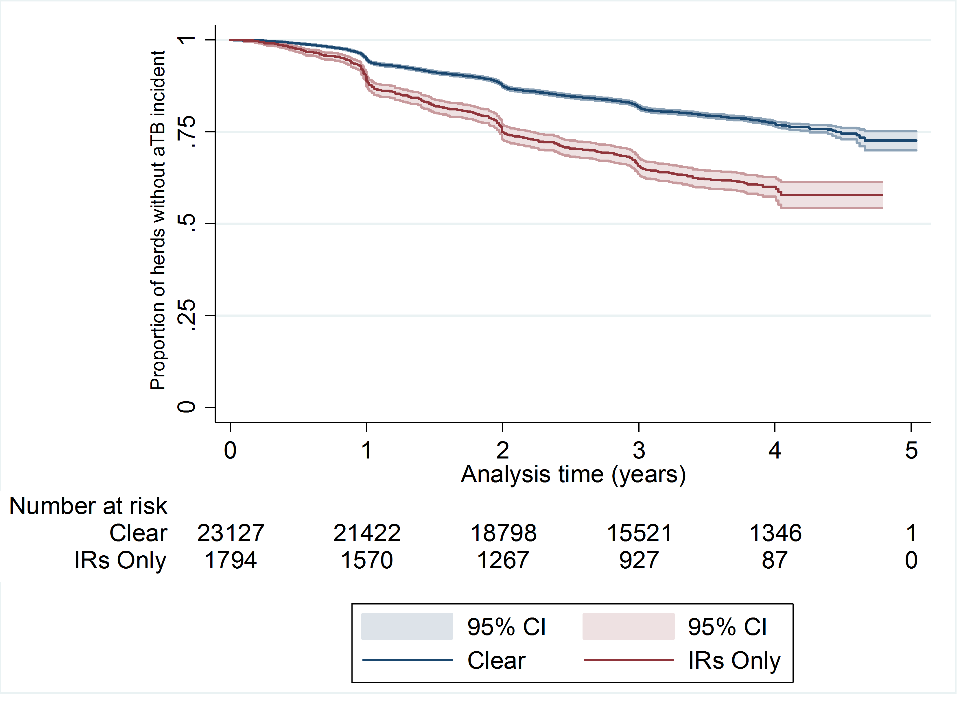


**Supplementary Figure 1.** Kaplan-Meier survival estimates for herds according to first WHT status in 2012 (N=24,921)

**Supplementary Table 3.** Results of the log-rank tests for equality of survivor functions

| **Variable** | **Chi-squared** | **P value** |
| --- | --- | --- |
| First WHT status in 2012 | 303.9 | <0.001 |
| Season in which 2012 WHT took place | 4.0 | 0.265 |
| Number of incidents in the previous 10 years | 1241.0 | <0.001 |
| Geographical risk area | 672.1 | <0.001 |
| Herd type | 628.7 | <0.001 |
| Herd size | 2292.4 | <0.001 |
| Annual rolling county-level incidence at end of 2012 | 707.1 | <0.001 |

## Assessment of the hazard of a subsequent incident among clear and IR only herds

There were strong associations between the hazard of a subsequent incident and each of the explanatory variables except for season in the univariable analysis (Supplementary Table 4). Factors found to be associated with an increased hazard were having an IR only test result at the 2012 WHT, a recent history of TB, being a dairy herd (compared to a beef herd), increasing herd size, and increasing county-level incidence. Herds in the edge area of England, and those in Wales, had a reduced hazard of a subsequent incident when compared to the high-risk area of England, as did herds classed as production type “other” compared with beef herds.

**Supplementary Table 4.** Results of the univariable Cox regression analysis of factors associated with the rate of subsequent incidents (N=28,022)

| **Variable** | **Level** | **HR^1^** | **95% CI^2^** | | **P value** | |
| --- | --- | --- | --- | --- | --- | --- |
| First WHT status in 2012 | Clear | *1.00* |  |  |  | |
|  | IRs only | 1.97 | 1.81 | 2.14 | <0.001 | |
| Season in which first WHT took place | Spring | *1.00* |  |  |  | |
|  | Summer | 1.01 | 0.93 | 1.11 | 0.764 | |
|  | Autumn | 1.03 | 0.96 | 1.11 | 0.394 | |
|  | Winter | 1.07 | 1.00 | 1.14 | 0.064 | |
| Number of incidents in the previous 10 years |  | 1.50 | 1.48 | 1.53 | <0.001 | |
| Geographical risk area | England high risk | *1.00* |  |  |  | |
|  | England Edge | 0.46 | 0.41 | 0.51 | <0.001 | |
|  | Wales | 0.47 | 0.44 | 0.50 | <0.001 | |
| Annual rolling county level incidence at end of 2012 |  | 1.07 | 1.07 | 1.08 | <0.001 | |
| Herd type | Beef | *1.00* |  |  |  | |
|  | Dairy | 2.02 | 1.90 | 2.14 | <0.001 | |
|  | Other | 0.44 | 0.30 | 0.63 | <0.001 | |
| Herd size | 1-10 | *1.00* |  |  |  | |
|  | 11-50 | 2.22 | 1.94 | 2.54 | <0.001 | |
|  | 51-100 | 3.79 | 3.31 | 4.33 | <0.001 | |
|  | 101-200 | 5.54 | 4.86 | 6.32 | <0.001 | |
|  | 201-300 | 7.35 | 6.37 | 8.48 | <0.001 | |
|  | >300 | 9.98 | 8.70 | 11.46 | <0.001 | |
| ^1^ Hazard ratio, ^2^ confidence interval | | | | | |  |

The initial multivariable Cox regression model included first WHT status in 2012, herd size, the number of incidents in the 10 years before the first WHT in 2012, herd type, geographical risk area and county-level incidence. The plot of the Cox-Snell residuals (Supplementary Figure 2) indicated that the model was a poor fit, and the plot of the deviance residuals over time (Supplementary Figure 3) revealed a number of observations that were not well fit by the model, particularly those herds with the shortest survival time. However, the Harrell’s C statistic was 0.74 indicating that the model correctly predicted the sequence of two observed failures 74% of the time. Assessment of the proportionality of the hazards using the log-minus-log plot (Supplementary Figure 4) indicated that the ratio of hazards varied less over time that the model reported in the accompanying manuscript. The Chi-squared test of the correlation between the Schoenfeld residuals of each variable and transformed time generated a p value <0.05 for herd size=51-100, county level incidence, herd type=other and risk area=Wales. This indicated that there was still some violation of the proportional hazards assumption. The correlation between the Schoenfeld residuals of first WHT status and transformed time was not significant (p=0.571) which indicated that the new entry date had resolved the lack of proportionality in the hazards relating to the IR re-test. Each of the four variables with significant correlation between the Schoenfeld residuals and transformed time were entered in to the model as an interaction with log time, and the models assessed using the likelihood ratio test and AIC.

**Supplementary Figure 2.** Plot of Cox-Snell residuals for the initial Cox regression

**Supplementary Figure 3.** Plot of deviance residuals for the initial Cox regression

**Supplementary Figure 3.** Log-minus-log survival plot for first WHT status adjusted for herd size, the number of incidents in the 10 years before the first WHT in 2012, herd type, county level TB incidence and geographical risk area

The final extended multivariable Cox regression model included first WHT status in 2012, herd size, the number of incidents in the 10 years before the first WHT in 2012, herd type and county level incidence as main covariates. Geographical risk area was included as a time-varying covariate, since it was no longer significant as a main covariate when included as an interaction with time. An additional time-varying coefficient representing the interaction between county level incidence and time was also included (Supplementary Table 5). The likelihood ratio test generated a p value <0.001, indicating that the final regression model differed significantly from a model containing no time-varying coefficients and including risk area as a main covariate.

As for the model using first WHT in 2012 as the entry date, the hazard of a subsequent incident was greater among IR only herds compared with herds that had a clear test result (after adjusting for herd size, testing following the 2012 WHT, recent history of TB, herd type, geographical risk area and county-level incidence) (Supplementary Table 5). The effect was smaller, which correlates with the finding reported in the accompanying manuscript that the hazard ratio decreased over time, but was still greater than 1.0 after the 60 re-test was passed.

**Supplementary Table 5.** Multivariable extended Cox regression model of factors associated with a subsequent incident, with time-varying coefficients

| **Variable** | **Level** | **HR^1^** | **95% CI^2^** | | **P value** | |
| --- | --- | --- | --- | --- | --- | --- |
| *Main covariates* |  |  |  |  |  | |
| First WHT status in 2012 | Clear | *1.00* |  |  |  | |
|  | IRs only | 1.19 | 1.10 | 1.30 | <0.001 | |
| Herd size | 1-10 | *1.00* |  |  |  | |
|  | 11-50 | 2.19 | 1.92 | 2.51 | <0.001 | |
|  | 51-100 | 3.57 | 3.12 | 4.09 | <0.001 | |
|  | 101-200 | 4.92 | 4.30 | 5.63 | <0.001 | |
|  | 201-300 | 6.32 | 5.45 | 7.33 | <0.001 | |
|  | >300 | 8.56 | 7.39 | 9.92 | <0.001 | |
| Number of incidents in the previous 10 years |  | 1.22 | 1.19 | 1.24 | <0.001 | |
| Herd type | Beef | *1.00* |  |  |  | |
|  | Dairy | 1.03 | 0.96 | 1.10 | 0.042 | |
|  | Other | 0.46 | 0.32 | 0.66 | <0.001 | |
| Annual rolling county level incidence at end of 2012 | 0-14.6 per 100 HYR^3^ | *1.00* |  |  |  | |
|  | >14.6 per 100 HYR^3^ | 1.08 | 1.06 | 1.09 | <0.001 | |
| *Time-varying covariates* |  |  |  |  |  | |
| Geographical risk area | England high risk | *1.00* |  |  |  | |
|  | England Edge | 0.99 | 0.87 | 1.12 | 0.820 | |
|  | Wales | 0.74 | 0.68 | 0.80 | <0.001 | |
| Annual rolling county level incidence at end of 2012 | 0-14.6 per 100 HYR^3^ | *1.00* |  |  |  | |
|  | >14.6 per 100 HYR^3^ | 0.98 | 0.97 | 1.00 | 0.006 | |
| ^1^ Hazard ratio, ^2^ confidence interval, ^3^ herd years at risk | | | | | |  |

# Conclusions

Using the revised entry date appeared to resolve the lack of proportionality in the hazards relating to the IR re-test, although some variables still violated the proportional hazards assumption. A significant difference in the hazard of a subsequent incident between clear and IR only herds was still observed. The results were aligned with the conclusion that there is a reduction in the hazard for IR only herds over time, but that the hazard of a subsequent TB incident is still higher among IR only herds than herds that tested negative to a whole herd test once the effect of re-testing has been removed. The same explanatory variables were found to be important in both model specifications.

However, a major concern with this analysis is that the IR cohort is disproportionately affected by changing the entry date, due to missing or inaccurate values within the subsequent clear test variable. As described in the results, 46% of IR only herds are lost from the analysis, compared with 15% of clear herds. This introduces a considerable bias to the analysis and makes it difficult to draw firm conclusions about the fate of IR only herds compared to clear herds after they get through the IR testing regime.
